# A Novel 4D Ultrasound Parenting Intervention for Substance Using Pregnant Women in Finland: Participation in Obstetric Care, Fetal Drug Exposure, and Perinatal Outcomes in a Randomized Controlled Trial

**DOI:** 10.1007/s10995-019-02773-w

**Published:** 2019-06-27

**Authors:** Heidi Jussila, Marjukka Pajulo, Eeva Ekholm

**Affiliations:** 1 Doctoral Programme of Clinical Investigation, Department of Child Psychiatry, University of Turku, 20014 Turku, Finland; 2 Department of Child Psychiatry, Turku University Hospital, Kiinamyllynkatu 4-8, PL 52, 20521 Turku, Finland; 3 Department of Child Psychiatry and The FinnBrain, University of Turku, 20014 Turku, Finland; 4 Department of Obstetrics and Gynecology, Turku University Hospital, Kiinamyllynkatu 4-8, PL 52, 20521 Turku, Finland; 5 Department of Obstetrics and Gynecology, University of Turku, 20014 Turku, Finland

**Keywords:** Prenatal care, Pregnancy, Intervention, Ultrasound, Substance use disorder

## Abstract

**Objectives:**

The aim of the study was to explore the effect of a new prenatal intervention on participation in obstetric care, fetal drug exposure, and perinatal outcomes among substance using pregnant women in Finland.

**Methods:**

The participants were 90 women referred to a hospital obstetric outpatient clinic due to current or recent substance use. The intervention group (n = 46) was offered three interactive ultrasounds at 24, 30 and 34 gestational weeks and a pregnancy diary accompanied by three prenatal infant mental health consultations. The intervention elements were designed to enhance parental mentalization and prenatal attachment. A randomized control group (n = 44) design was used. All participants were offered treatment-as-usual in the obstetric tertiary setting. Medical record data and meconium toxicology were analyzed.

**Results:**

The retention rate in the whole sample was 89%. Retention was higher in the intervention group (96% vs. 82%, *p *< 0.05), of which 74% attended all three ultrasound sessions. However, the pregnant women in the intervention group participated less often in all the scheduled obstetric standard care visits (59% vs. 83%, *p *= 0.02). Fetal drug exposure and perinatal outcomes were similar in both groups. Within the whole sample, 13% of the neonates were preterm, 12% small for gestational age and 7% had exposure to drugs.

**Conclusions for Practice:**

Retention in the intervention was very good. Watching the fetus with parenting focus seemed to motivate these high-risk women. Interestingly, the pregnant women in the intervention group tended to prefer the intervention sessions to the routine care. Clinical implications of this finding are discussed.

***Trial Registry*:**

The trial registration number in ClinicalTrials.gov: NCT03413631.

## Significance

Prenatal substance use constitutes a severe public health concern. Fetal substance exposure is a preventable risk factor in the intergenerational chain of inequality and disadvantage determining the child’s health at birth and short- and long-term outcome. However, prenatal interventions developed for women with substance use disorder are scarce. This randomized controlled study explored the effect of a novel 4D ultrasound parenting intervention on participation in obstetric care, fetal drug exposure and perinatal outcomes among substance using pregnant women. Retention of these high-risk patients with this new approach was very good, but a decrease in fetal exposure or improved perinatal outcomes were not observed.

## Introduction

Substance use during pregnancy constitutes a growing public health problem. In the United States, 9.4% of pregnant women self-report current alcohol use and 5.4% illicit drug use (Substance Abuse and Mental Health Services Administration 2014). The worldwide prevalence rate of fetal alcohol spectrum disorder was found to be higher than estimated so far, around 23 per 1000 live births (Roozen et al. 2016). In many countries, increased use of prescription opioids has been described as epidemic, also among women who are of a reproductive age or pregnant (Ollgren et al. 2014; Reddy et al. 2017). Recently, the number of illicit drug users, as well as the proportion of reproductive-aged women among them, has increased in Finland (Ollgren et al. 2014).

Prenatal smoking, heavy drinking, and use of illicit drugs often lead to adverse neonatal outcomes, especially preterm birth and a small weight for gestational age (Forray and Foster 2015; Kotelchuck et al. 2017; Ludlow et al. 2004). Prescription opioid use in pregnancy is known to cause perinatal complications, of which the most significant one is neonatal abstinence syndrome (Behnke et al. 2013; Forray and Foster 2015; Reddy et al. 2017). Prenatal substance use is a preventable risk factor in the intergenerational chain of inequality and disadvantage determining the child’s health at birth and developmental outcomes (Aizer and Currie 2014; Behnke et al. 2013; Forray and Foster 2015). Nevertheless, prenatal psychosocial interventions for women suffering from substance use disorders are scarce, and studies have rarely evaluated obstetric, neonatal or child outcomes (Forray and Foster 2015; Haug et al. 2014; Neger and Prinz 2015; Terplan et al. 2015).

Substance use disorders have a strong association with inadequate prenatal care (Kotelchuck et al. 2017; Roberts and Pies 2011). In addition, an increased number of emergency visits and hospitalizations but less participation in preventive care has been reported among substance using pregnant women (Kotelchuck et al. 2017). One specific problem is the question of how to engage these high-risk patients in prenatal healthcare (Kotelchuck et al. 2017; Roberts and Pies 2011).

Caregiving is a driving motivational force for recovery in substance use disorders (Jessup et al. 2014). Stronger maternal–fetal attachment has been linked with favorable prenatal health practices and better neonatal outcomes among low-income women (Alhusen et al. 2012). According to previous studies, maternal–fetal attachment can be strengthened by ultrasound imaging in normative pregnant women (Yarcheski et al. 2009). A higher capacity for parental mentalization has been found to associate with a lower risk of relapsing into substance use (Pajulo et al. 2012), as well as more sensitive caregiving (Camoirano 2017) among parenting women with substance use disorders. Prenatal parental mentalization refers to the parent’s ability to reflect on the fetus as a separate individual with developing capacities and emerging personality (Pajulo et al. 2015). Mentalization focus has specific intervention potential in high-risk groups, and the parental mentalization capacity of substance using mothers can be enhanced with interventions (Camoirano 2017).

A new prenatal intervention was designed for substance using women in public health care in Finland. The intervention aimed to enhance maternal–fetal attachment by focusing on parental mentalization using interactive 4D ultrasound imaging, and a week-by-week pregnancy diary. The objective was to assess the effect of the intervention on (1) fetal drug exposure detected by meconium testing, (2) perinatal outcomes and (3) participation in obstetric care. A randomized and controlled design was used. The intervention group was hypothesized to display better perinatal outcomes and participation in obstetric care, and less fetal drug exposure.

## Methods

### Participants

The study was conducted at the obstetric outpatient clinic for substance using pregnant women at Turku University Hospital between October 2011 and November 2014. The women were referred from primary health care due to (1) documented or self-reported illicit drug use, abuse of prescription medication or alcohol within 3 years prior to or during the present pregnancy, (2) and/or a sum score of ≥ 3 points on TWEAK alcohol screening (Russell 1994). At admission, a psychiatric nurse offered all the eligible women an opportunity to participate. The inclusion criteria were a pregnancy duration of < 22 gestational weeks (gwks) at referral, and a singleton pregnancy. A written informed consent was obtained from all individual participants included in the study. The participants were randomized into the intervention and control groups using a computer-generated block-randomization with a block size of four, and separate blocks for women in buprenorphine substitution treatment. Of all the eligible women (n = 126) referred, 75% consented to participate and were randomized into the intervention (n = 47) and control groups (n = 48). The participants were compensated with a 20 euros gift card at 35 gwks, with the gift being targeted to the child’s needs.

The patients in both groups were scheduled visits at the obstetric outpatient clinic for pregnancy follow-up including clinical obstetric examinations, laboratory testing, and assessments of fetal growth and well-being with ultrasound imaging. Urine samples were collected for drug screening. The pregnant women were interviewed by the psychiatric nurse and the social worker on substance abuse, health and social situation based on the European Addiction Severity Index-questionnaire (EuropASI) (Kokkevi and Hartgers 1995). The patient was referred to addiction treatment and psychiatric care, if needed. Based on Finnish legislation, Child Welfare authorities were already informed in the prenatal phase when substance use in pregnancy was identified. A joint meeting was scheduled for the patients in the third trimester.

### Intervention

The intervention was based on the interactive use of 4D ultrasound imaging and the pregnancy diary. Both elements were designed to enhance prenatal parental mentalization and maternal–fetal attachment. The intervention method has been described earlier (Pajulo et al. 2016). The control group received comprehensive treatment-as-usual in tertiary obstetric care. The protocol is introduced in Table 1.

**Table 1:** The protocol of intervention and treatment-as-usual in the hospital obstetric outpatient clinic

	< 22 gwks	< 24 gwks	24 gwks	30 gwks	34 gwks	35 gwks
Standard care for the intervention and control groups	EuropASI interviews by a psychiatric nurse and a social worker Urine drug screening Submission of a Child Welfare Notification	Obstetric assessments including fetal ultrasounds				A joint meeting with The Child Welfare authorities
The study protocol for the intervention and control groups	Information Recruitment Literal consent	Randomization Background data Questionnaires				Questionnaires 20 Euros gift card
The intervention protocol for the intervention group		Pregnancy diary	4D US session Diary session	4D US session Diary session	4D US session Diary session	

*gwks* gestational weeks, *EuropASI* The European Addiction Severity Index-questionnaire, *4D* four-dimensional ultrasound

The intervention group was offered three interactive 4D ultrasounds at 24, 30 and 34 gwks. The sessions lasted for 20–30 min, and were performed by an obstetrician and an infant mental health professional working in collaboration. During the sessions, the women were encouraged to observe the fetus and to reflect on their own emotions towards the fetus and becoming a parent for this particular child. The session started with watching the fetus according to the mother’s wish. How the fetus looked, his/her facial expressions, behavior, initiatives for interaction and developing capacities were highlighted. The aim was to arouse the woman’s active interest in the perspective of her child as a separate individual, and to keep the fetus in the mother’s mind. Further, the intervention group received a mentalization-focused pregnancy diary and three sessions with the infant mental health professional to work with it. The diary contains an information spread sheet for each pregnancy week with psychoeducation about pregnancy, fetal development and recommended health practices. The key elements of the diary are questions and small tasks evoking the woman to consider her baby’s point of view and her own experiences about becoming a parent (Pajulo et al. 2011). The sessions with the infant mental health professional were arranged to give the participant the possibility to reflect on her thoughts and experiences inspired by the diary.

### Data Collection and Measures

As a part of the clinical assessment, the psychiatric nurse and the social worker interviewed the participants at admission. The interviews were based on items in the European Addiction Severity Index-questionnaire concerning somatic and mental health, substance use, employment, income, legal status and close relationships (Kokkevi and Hartgers 1995). Data regarding maternal mental health was based on the interview and hospital medical records. Socio-demographic data was obtained at baseline with a questionnaire. Standardized self-report measures for the assessment of maternal mental health and prenatal parenthood were administered before the intervention (< 24th gwks) and after the intervention (> 34th gwks) (Pajulo et al. 2016).

After birth, the meconium was analyzed qualitatively with liquid chromatography–tandem mass spectrometry (LC–MS/MS) for tramadol, morphine, 6-monoasethylmorphine, codeine, oxycodone, methadone, buprenorphine, norbuprenorphine, amphetamine, methamphetamine, 3,4-methylenedioxymethamphetamine, 3,4-methylenedioxyamphetamine and a metabolite of tetrahydrocannabinol (THC-COOH). Further, the meconium sample was screened qualitatively for more than 600 different pharmacological agents and 300 tranquilizers or illicit drugs with ultra-high performace liquid chromatography–quadrupole time-of-flight mass spectrometry (UHPLC–QTOF/MS). Meconium testing indicates intrauterine drug exposure during the second and the third trimester of pregnancy (Wabuyele et al. 2018).

The data regarding participation in obstetric care and perinatal outcomes was obtained from the hospital medical records. Small for gestational age (SGA) was defined as a birth weight more than 2 standard deviations (< 2SDs) below the population mean weight for gestational age, and preterm as a birth before 37 gwks. Finnegan scoring was used for assessment of neonatal withdrawal symptoms (Finnegan et al. 1975).

### Ethical Approval

All procedures were in accordance with the ethical standards of the Responsible Committee on Human Experimentation and with the Helsinki Declaration of 1964 and its later amendments. The research was approved by the Joint Ethics Committee of the University of Turku and The Hospital District of Southwest Finland on 14th of June 2011. The trial was registered retrospectively in the ClinicalTrials.gov (the reference number NCT03413631).

### Statistical Analyses

The sample size and power analysis were calculated based on the primary outcome of the study, prenatal depressive symptoms (EPDS), not regarding the outcomes explored in the current study.

Comparisons between the intervention and control group in nominal variables were done using chi square test or Fisher’s exact test, as appropriate. The continuous variables were compared between the groups with an independent samples *t* test or a Mann–Whitney *U* test, as appropriate. The magnitudes of the effect size for the non-parametric comparisons were calculated using a formula ($r = Z \div \surd N$) (Field 2013). Statistical analyses were done with SPSS version 24, and a *p*-value of < 0.05 was considered as significant.

## Results

### Attrition

**Fig. 1:**
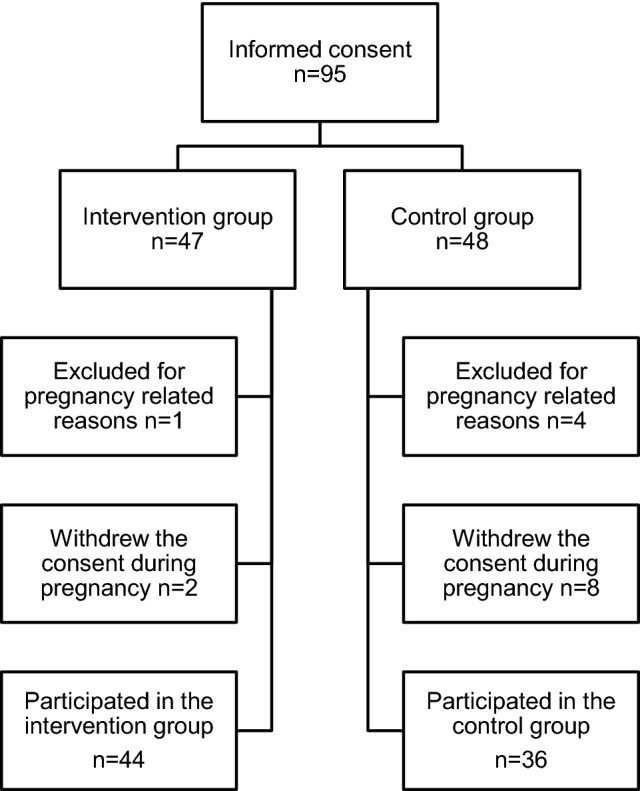
Attrition

Ninety-five pregnant women gave informed consent. Five participants were excluded after randomization due to pregnancy related reasons (twin pregnancy, miscarriage or induced abortion). The 90 women included in the study were recruited at a median of 13 (range 7–22) gwks. Ten women (11%) discontinued their participation during pregnancy. Eight of these women were allocated to the control group, and they withdrew consent immediately after randomization. Attrition in the control group was significantly higher than in the intervention group [18% (8/44) vs. 4% (2/46), *p *= 0.047] (Fig. 1). Three women in the intervention group moved to a location outside the hospital district.

### Sample Characteristics

Descriptive data of the patients is presented in Table 2. The mean age of the participants was 25 (SD 5.5) years. The pregnancy was unintended in 70% of the cases, and 71% of the women were nulliparous. The median length of substance use was 4 (range 0–28) years, 81% of the participants reported illicit drug use, and 49% both drug and alcohol abuse. Twenty-eight percent of the participants reported somatic comorbidities, most commonly respiratory, neurological, musculoskeletal or thyroid illnesses. In addition, 27% of participants were seropositive for hepatitis C. The sample characteristics were similar in both groups, indicating successful randomization.

**Table 2:** Descriptive data of the participants

	All	Intervention	Control			
	*n/N* (%)	*n/N* (%)	*n/N* (%)	df	*χ*^*2*^	*p*
Socio demographic background						
Only basic education (≤ 9 years)	44/80 (55)	24/44 (55)	20/36 (56)	2	0.06	.97
Low monthly income (< 1000 euros)	44/78 (56)	25/45 (56)	19/33 (58)	1	0.03	.86
Crimes in background	35/85 (41)	19/45 (42)	16/40 (40)	1	0.04	.84
Children in foster care	14/87 (16)	5/45 (11)	9/42 (21)	1	1.71	.19
An intimate relationship	60/79 (76)	28/41 (68)	32/38 (84)	1	2.74	.10
Living situation at recruitment				3	2.71	.44
With the child’s father	47/86 (55)	21/45 (47)	26/41 (63)			
Alone	29/86 (34)	18/45 (40)	11/41 (27)			
With someone else	4/86 (5)	2/45 (4)	2/41 (5)			
With parents	6/86 (7)	4/45 (9)	2/41 (5)			
Child’s father has a history of substance abuse	54/82 (66)	32/43 (74)	22/39 (56)	1	2.95	.09
Substance abuse and comorbidity						
Self-reported substance use (ever)						
Alcohol	62/90 (69)	33/46 (72)	29/44 (66)	1	0.36	.55
Cannabis	48/90 (53)	25/46 (54)	23/44 (52)	1	0.04	.84
Amphetamine, other stimulants	42/90 (47)	21/46 (46)	21/44 (48)	1	0.04	.84
Benzodiazepines	34/90 (38)	20/46 (43)	14/44 (32)	1	1.30	.25
Buprenorphine	26/90 (29)	14/46 (30)	12/44 (27)	1	0.12	.74
History of intravenous substance abuse	40/85 (47)	21/46 (46)	19/39 (49)	1	0.08	.78
Smoking during pregnancy	79/87 (91)	43/46 (93)	36/41 (88)			.47^a^
Reduction of smoking in pregnancy	68/81 (84)	38/44 (86)	30/37 (81)	1	0.41	.52
Cessation of smoking in pregnancy	22/74 (30)	14/40 (35)	8/34 (24)	1	1.15	.28
Psychiatric comorbidity	69/87 (79)	36/46 (78)	33/41 (80)	1	0.07	.79
Somatic comorbidity	24/87 (28)	10/46 (22)	14/41 (34)	1	1.67	.20
Psychopharmacological medication	31/77 (40)	15/41 (37)	16/36 (44)	1	0.49	.48
Buprenorphine substitution for opioid addiction	16/87 (18)	9/46 (20)	7/41 (17)	1	0.09	.76
Residential care during pregnancy	14/77 (18)	9/41 (22)	5/36 (14)	1	0.84	.36
Out-patient psychiatric treatment for addiction	52/78 (67)	27/42 (64)	25/36 (69)	1	0.23	.63
Out-patient psychiatric treatment	16/77 (21)	9/41 (22)	7/36 (19)	1	0.07	.77

*N* information available, *n* a number of affirmative responses

^a^Fisher’s exact test

### Intervention Attendance

The median number of attended ultrasound sessions was 3 (range 0–3) and of diary meetings 1 (range 0–3). The attendance rate at the interactive ultrasound sessions was 96% (44/46), and 74% (34/46) of the participants in the intervention group attended all three ultrasound sessions. Of those 12 women not attending all the ultrasound sessions, four delivered prematurely, two withdrew from the study and six did not attend because of logistic reasons (moving to another hospital district or problems in scheduling by the research group). The attendance rate at the diary meetings was 65% (30/46).

### Participation in Obstetric Care and Perinatal Outcomes

The number of prenatal obstetric consultations, emergency department visits, and hospital admissions was equal in both groups (Table 3). However, all the scheduled clinical visits in the context of treatment-as-usual were attended by 59% of the patients in the intervention group compared to 83% in the control group [χ^2^(1, *N *= 80) = 5.54*, p *= 0.02]. Forty-eight percent of the women suffered from pregnancy complications, such as obesity, gestational diabetes, hypertension, pre-eclampsia or infections. Seventy-one percent of the women had an uncomplicated vaginal delivery. A vacuum extraction was performed in 11% and a cesarean section in 18% of the participants. The median birth weight was 3310 (range 645–4870) g. Thirty-five percent of the neonates were treated at the neonatal intensive care unit. One neonate in the intervention group died from asphyxia. The median pH of umbilical artery at birth was 7.25 (range 7.11–7.47). The perinatal outcomes were similar in both groups (Table 3).

**Table 3:** Participation in prenatal care and perinatal outcomes in the study population

Participation in prenatal hospital care	*N*	Mean	SD	Median	Range	*p*	*r*^d^
All consultations at the obstetric outpatient clinic							
All	75	8.3	3.7	8.0	3 to 23		
Intervention	40	8.1	3.5	8.0	3 to 22		
Control	35	8.4	4.0	8.0	3 to 23	0.94^a^	0.01
Emergency consultations at the obstetric outpatient clinic							
All	75	1.1	1.4	1.0	0 to 7		
Intervention	40	1.0	1.2	1.0	0 to 5		
Control	35	1.3	1.6	1.0	0 to 7	0.39^a^	0.10
Prenatal hospital admissions (obstetric care)							
All	73	0.4	1.0	0	0 to 7		
Intervention	38	0.3	0.8	0	0 to 4		
Control	35	0.6	1.2	0	0 to 7	0.11^a^	0.19
Prenatal hospital care (days) at the obstetric department							
All	73	1.4	4.5	0	0 to 31		
Intervention	38	0.6	1.5	0	0 to 8		
Control	35	2.3	6.3	0	0 to 31	0.08^a^	0.20
Emergency department visits							
All	71	0.5	0.9	0	0 to 4		
Intervention	38	0.6	1.1	0	0 to 4		
Control	33	0.3	0.6	0	0 to 2	0.27^a^	0.13

	*N*	*n*	%	*p*	Cramer’s *ϕ*
Participants with withdrawn visits at the obstetric clinic					
All	80	24	30		
Intervention	44	18	41		
Control	36	6	17	0.02^b^*	0.26

Perinatal outcomes	*N*	Mean	SD	Median	Range	*p*	*r*^d^
Gestational weeks at delivery							
All	80	38.9	2.6	39.5	25 to 42		
Intervention	44	39.0	2.8	40.0	25 to 41		
Control	36	38.8	2.5	39.0	28 to 42	0.19^a^	0.15
Birth weight standard deviation (SD)							
All	76	− 0.7	1.1	− 0.8	− 3.2 to 2.2		
Intervention	41	− 0.7	1.0	− 0.6	− 3.2 to 1.6		
Control	35	− 0.7	1.2	− 1.0	− 2.5 to 2.2	0.72^a^	0.04
Head circumference standard deviation (SD)							
All	73	− 0.5	0.9	− 0.6	− 2.8 to 2.0		
Intervention	38	− 0.6	0.8	− 0.8	− 2.6 to 1.6		
Control	35	− 0.4	1.0	− 0.5	− 2.8 to 2.0	0.28^a^	0.13
5 min Apgar score							
All	75	8.8	1.0	9.0	3 to 10		
Intervention	41	8.7	1.2	9.0	3 to 10		
Control	34	8.9	0.6	9.0	8 to 10	0.66^a^	0.05
Length of hospital stay, days (child)							
All	78	7.9	12.7	4.5	0 to 90		
Intervention	43	9.3	14.9	5.0	0 to 90		
Control	35	6.1	9.3	4.0	2 to 58	0.21^a^	0.14

	*N*	*n*	%	*p*	Cramer’s *ϕ*
Small for gestational age (< 2SD)					
All	76	9	12		
Intervention	41	4	10		
Control	35	5	14	0.73^c^	0.07
Low birth weight (< 2500 g)					
All	80	9	11		
Intervention	44	5	11		
Control	36	4	11	1.00^c^	0.004
Preterm (< 37 gwks)					
All	80	10	13		
Intervention	44	6	14		
Control	36	4	11	1.00^c^	0.04
Neonatal withdrawal symptoms					
All	76	12	16		
Intervention	40	4	10		
Control	36	8	22	0.21^c^	0.17

Difference between the intervention and the control group: ^a^Mann Whitney *U* test, ^b^Pearson Chi Square test, ^c^Fisher’s exact test, ^d^Effect size *r* = 0.1 is referring to small effect, *r* = 0.30 is referring to medium effect, and *r* = 0.5 is referring to large effect (Field 2013)

*N* data available, *n* a number of relevant cases

*p ≤ .05

### Fetal Drug Exposure

Meconium testing was available for 89% (71/80) of the neonates, 94% (34/36) in the control and 84% (37/44) in the intervention group. Based on the meconium testing, 7% (5/71) of the newborns were exposed to illicit drugs or non-medical use of prescription drugs. A difference was not displayed in fetal drug exposure, as the meconium was screened positive for illicit drugs in 3% (1/37) of the newborns in the intervention group and 12% (4/34) in the control group (Fisher’s exact test, *p *= 0.18).

## Discussion

The objective was to explore the effect of a novel prenatal parenting intervention on participation in obstetric care, fetal drug exposure and perinatal outcomes among substance using women. Retention of these high-risk pregnant women was found to be very good in the mentalization-focused intervention using interactive 4D ultrasound and the pregnancy diary. The high attendance at the ultrasound sessions is to be especially highlighted. Interestingly, the participants in the intervention group tended to prefer the intervention sessions to the standard obstetric care visits. Contrary to the hypothesis, the intervention did not affect fetal drug exposure or the perinatal outcomes.

The participants comprised a high-risk sample with severe substance use problems and accumulation of psychosocial and health related risks (see Table 2). The retention rate of 89% in the whole sample and, especially the 96% in the intervention group, can be considered very good, also when compared to previous studies. In prenatal psychosocial interventions for substance using women, such as contingency management and the motivational interview based approach, the retention rates have varied between 28 and 100% (Terplan et al. 2015). Moreover, a recent systematic review reported a considerable attrition of 22–33% even in medication-assisted treatment programs for opioid dependent pregnant women (Zedler et al. 2016). Parenting interventions for substance using mothers have yielded a treatment retention rate of 16–100% and a study retention rate of 33–93% (Neger and Prinz 2015). Except for one study with a sample size less than ten, our study showed better patient retention than other previous studies exploring the effects of psychosocial interventions for pregnant women with illicit drug use (Terplan et al. 2015).

Only 4% of the women randomized to the intervention group discontinued, compared to 18% of the control group. In the control group, the participants who dropped out discontinued immediately after randomization, when they were allocated to the group not receiving the intervention. The intervention group was offered 4D ultrasounds and specific attention and theoretically grounded support for their prenatal parenting (Pajulo et al. 2016). In the current study, the high attendance at the ultrasound sessions has been emphasized. We can therefore speculate that this very good retention was due to the potential of the intervention approach, especially the parenting-focused 4D interactive ultrasounds, to engage these high-risk patients. The pregnancy diary may also have contributed to the study retention by stimulating interest towards the fetus, although the attendance at the separate diary visits was not as high. Discussions about thoughts aroused by the diary may have been challenging for these women, who are often verbally scarce (Pajulo et al. 2012), and weak in insight (Goldstein et al. 2009) and self-mentalization (Camoirano 2017). Scheduling the diary visits with the ultrasound sessions might help to increase the attendance rate. The intervention was multifaceted and, thus the contribution of each component to the study retention could not be explored separately. Unfortunately, the difference in attrition may also reflect an attrition bias and diminish the experimental validity of the study.

Among the intervention group, we observed very good study retention but, unexpectedly, weaker compliance to standard care. The number of emergency visits, obstetric consultations and hospitalizations was similar in both groups but, contrary to our hypothesis, a higher number of patients in the intervention group missed standard antenatal visits. This finding should be considered when implementing the intervention in clinical practice. Based on the very good study retention and favorable attendance at the ultrasound session, the pregnant women found the intervention approach attractive and acceptable. We speculate that more disadvantaged patients may have engaged in the intervention because of the interest towards the intervention elements, especially the interactive 4D ultrasounds. Possible attrition bias may have been reflected in our findings concerning adherence to treatment-as-usual; in addition to which, the studied intervention may also have interfered with their compliance to standard care. The finding may be explained by the different method of encountering the substance using pregnant women in the intervention sessions compared to standard antenatal visits. Our intervention aimed to enhance their role as a caregiver and to empower them in prenatal parenting and attachment in an interactive and reflective way, strengthened by the concrete 4D visualization of the fetus combined with the parenting focus. We suggest that the patients in the intervention group may have prioritized the study visits and the intervention approach over the standard care visits. Previous research has established that vulnerable women should be supported in their parenting role already in pregnancy (Glover and Capron 2017). The present intervention aims at enhancing parenting, which constitutes the most important protective environmental factor for child development after birth (Wlodarczyk et al. 2017). Further research would be valuable to assess whether the 4D parenting intervention influences the early mother–baby relationship. The interest of the substance using pregnant women towards the current intervention suggests that this approach is feasible in this high-risk population, and might be beneficial to integrate into standard obstetric care.

Meconium testing is considered to be the golden standard for detecting fetal drug exposure (Wabuyele et al. 2018). Unfortunately, objective means for detecting prenatal alcohol exposure are still inadequate (McQuire et al. 2016). The number of positive meconium tests was relatively small considering the severity of problems related to substance use in the study population, only 7% of the newborns were exposed to drugs. Contrary to the hypothesis, the group difference was not observed in fetal drug exposure. Previous studies on prenatal psychosocial interventions for women using illicit drugs have also failed to display greater abstinence in comparison to control conditions (Terplan et al. 2015). A few studies concerning brief interventions and integrated treatment approaches for pregnant women with substance use disorders have shown some promise in reducing fetal substance exposure (Farr et al. 2014; Haug et al. 2014; McLafferty et al. 2016). However, the problem remains that substance using pregnant women rarely receive adequate prenatal care (Kotelchuck et al. 2017).

The high-risk profile of the participants was reflected in the perinatal outcomes that showed a high rate of prematurity (13%), a low birth weight (11%) and perinatal mortality due to one asphyxia-related death. The Medical Birth Register shows that around 5.9% of neonates are born preterm, 4.4% have a low in birth weight and perinatal mortality is four per thousand in Finland (Vuori and Gissler 2016). Our findings on the poor perinatal outcomes among substance using women are in line with previous studies (Forray and Foster 2015; Kotelchuck et al. 2017; Ludlow et al. 2004). The perinatal outcomes were similar in both groups. Also, previous studies on prenatal psychosocial interventions have failed to display a difference in neonatal outcomes in comparison to comprehensive standard care among pregnant women with illicit drug use (Terplan et al. 2015). Prenatal treatment for substance use disorders may improve neonatal outcomes, but many substance using pregnant women do not adhere to treatment (Kotelchuck et al. 2017).

Not gaining statistically significant differences in perinatal outcomes and fetal drug exposure between the groups may possibly be due to the small sample size that was only able to detect moderate to large treatment effects. Further research should be undertaken to investigate the efficacy of the intervention with a larger sample. Although the study was randomized and the sample characteristics did not reveal any differences between the groups at baseline, the balance of confounding factors may have been different in the study groups due to the higher attrition in the control group. Possible retention of the more disadvantaged patients in the intervention group (attrition bias) may have compromised the detection of the intervention efficacy. The negative findings regarding the efficacy of the intervention may also simply result from the intervention not being effective enough within this very high-risk sample. The number of positive meconium drug tests was small, and conclusions concerning the effect of the intervention on fetal exposure should be drawn cautiously. Meconium drug testing may not be able to capture all the benefits of the intervention on maternal health practices. Further, all the participants were treated in the tertiary obstetric unit, and most of them were also engaged in other treatment modalities. High accessibility to medical care is associated with favorable neonatal outcomes (Aizer and Currie 2014) as is attending substance abuse treatment during pregnancy (Kotelchuck et al. 2017). Probably all the prenatal services have contributed to the outcomes and, thus the benefits of the studied intervention might have been difficult to differentiate in this setting. In addition, participating in the clinical trial per se may have had a favorable effect on both groups (Nijjar et al. 2017).

The strengths of the study are the novel routes used for prenatal intervention, the randomized and controlled design conducted in public health care and implemented into clinical setting with a multidisciplinary approach, and the multiple sources for data collection. The sample size was relatively small, attrition was higher in the control group and only meconium testing was used as the outcome for fetal substance exposure; all of which are considered limitations.

The study demonstrated that the retention of substance using pregnant women in the novel parenting intervention using 4D ultrasound was very good. High compliance to the current study has clinical implications, because in clinical practice much effort is required to engage these high-risk patients in prenatal care (Kotelchuck et al. 2017; Roberts and Pies 2011). Watching the baby with a parenting focus seemed to motivate these high-risk women, although we were not able to confirm the effect of the intervention on fetal drug exposure and the perinatal outcomes. Interestingly, the pregnant women in the intervention group may have preferred the intervention sessions to standard obstetric care. We speculate that this novel intervention approach combining a parenting focus with interactive 4D ultrasound visualization of the fetus, engenders an experience of meaningful encounters for these high-risk pregnant women.

## Abbreviations

4D: Four-dimensional
gwks: Gestational weeks
